# Effect of a Salt Reduction Campaign in a Community Clinic: A Retrospective Study

**DOI:** 10.7759/cureus.107518

**Published:** 2026-04-22

**Authors:** Hiroshi Sunagawa, Misa Chinen, Shota Miyagi, Kaoru Gaja, Kae Sunagawa, Misaki Ikema, Norifumi Kamiya, Sumito Sunagawa, Isao Komesu, Atsushi Sakima

**Affiliations:** 1 Internal Medicine Department, Sunagawa Medical Clinic, Uruma, JPN; 2 Health Administration Center, University of the Ryukyus, Nishihara, JPN

**Keywords:** community clinic, hypertension, noncommunicable disease, retrospective study, salt reduction campaign

## Abstract

Background: Excessive sodium chloride (salt) intake is a risk factor for noncommunicable diseases, such as hypertension, and initiatives to reduce salt intake are essential. This study assessed whether a salt reduction campaign reduced salt intake in outpatients attending a community clinic.

Methods: Of 2,429 outpatients whose baseline estimated salt intake was measured using spot urine during the campaign conducted from February 1 to June 30, 2018, 655 outpatients (42.4% female, mean age of 61.6 ± 13.1 years) with a baseline salt intake of ≥10 g/day and ≥2 salt intake measurements within six months of the campaign were included. During the campaign, healthcare providers displayed information on low-salt foods and a poster depicting the association between excess salt intake and hypertension, and encouraged outpatients to measure their salt intake. The primary outcome was the change in salt intake.

Results: Salt intake decreased from 11.6 ± 1.69 to 9.4 ± 2.44 g/day (p < 0.001), body mass index (BMI) decreased from 27.4 ± 5.0 to 27.2 ± 5.0 kg/m² (p < 0.001), and systolic blood pressure (SBP) decreased from 132.3 ± 13.0 to 131.1 ± 13.5 mmHg (p = 0.028) in this population. Correlation analysis of the factors associated with changes in salt intake identified changes in BMI (r = 0.192, p < 0.001) and SBP (r = 0.176, p < 0.001). Estimated glomerular filtration rate decreased slightly; however, no clinically significant renal events were identified.

Conclusion: This simple campaign represents a practical strategy to reduce salt intake in routine clinical practice.

## Introduction

Excessive sodium chloride (salt) intake increases the risk of hypertension and cardiovascular disease [1-5]. Lifestyle modifications, including salt reduction, are fundamental to the prevention and management of these conditions [1,6]. Salt reduction is particularly important in Japan, where high-salt diets are common [1,7]. According to the 2023 National Health and Nutrition Survey in Japan, mean daily salt intake remains high at 9.8 g (10.7 g in men and 9.1 g in women), showing no significant change over the past decade [8]. These levels exceed the targets of the Dietary Reference Intakes for Japanese 2025 (<7.5 g/day for men and <6.5 g/day for women) [9] and the recommendation of the Japanese Society of Hypertension Guidelines 2025 (<7.5 g/day for patients with hypertension) [1]. They also remained well above the WHO guidelines of <5 g/day [10].

Salt-reduction strategies may target both individuals and populations. Individual-level approaches include dietary interventions with reduced-salt or substitute foods, nutrition education provided by registered dietitians or healthcare professionals, and self-monitoring of the urinary sodium-to-potassium ratio or sodium excretion [1]. Population-level interventions include national or municipal health campaigns and group-based programs implemented in societies [11-13]. Meta-analyses of randomized controlled trials (RCTs) have confirmed the effectiveness of both individual-level [14-16] and population-based salt reduction interventions [17]. In Japan, several population-based salt reduction programs targeting communities [18-20], families [21,22], and workplaces [23,24] have been implemented and evaluated to demonstrate their effectiveness in reducing salt intake. Among these, community-based salt reduction campaigns conducted by Iso et al. demonstrated reductions in both dietary salt intake and blood pressure among residents [18-20]. However, to our knowledge, evidence regarding such campaigns in clinical settings remains limited worldwide [17]. Therefore, we examined whether a community clinic-based campaign could reduce estimated salt intake among outpatients and explored the associated factors.

The primary objective of this study was to assess whether a community clinic-based salt reduction campaign reduced estimated salt intake among outpatients. The secondary objective was to explore clinical and anthropometric correlates of changes in salt intake. We hypothesized that the campaign would be associated with a reduction in salt intake.

## Materials and methods

Study design and participants

This retrospective study was approved by the Ethics Committee for Life Science and Medical Research at the University of the Ryukyus, Nishihara, Japan (Approval No. 25-2532-00-00-00) and conformed to the principles of the Declaration of Helsinki (2024).

Among 2,429 outpatients whose estimated salt intake was measured by spot urine testing during a salt reduction campaign at Sunagawa Medical Clinic between February and June 2018, we included patients who met the following criteria: (1) baseline estimated salt intake ≥10 g/day, (2) at least two measurements of estimated salt intake within six months during the campaign period, and (3) availability of complete clinical data. Patients with missing data or a history of bariatric surgery or cancer surgery were excluded. A total of 655 patients were included in the final analysis.

During the campaign, the clinic displayed low-salt recipes, reduced-salt products, and food models illustrating high-salt foods. Urine samples were collected as spot urine samples during routine outpatient visits. The timing of urine collection (e.g., fasting state or time of day) was not standardized, as samples were obtained under usual clinical practice conditions. Estimated salt intake calculated from spot urine samples was fed back to patients during consultations with attending physicians. In addition, upon patient request, registered dietitians provided individualized counseling using a salt exchange table provided by the Japan Society for the Prevention of Severe Chronic Disease. Patients were followed at routine outpatient visit intervals, approximately every two to three months. During the campaign period, educational materials were freely accessible to all patients during clinic hours without restrictions on use. Individualized dietary counseling by registered dietitians was generally provided upon patient request.

Study procedure and outcomes

Information on the medical diagnoses of obesity, diabetes mellitus, dyslipidemia, and chronic kidney disease (CKD) was also collected from the electronic medical records. Obesity was defined as body mass index (BMI) of ≥25 kg/m²; diabetes mellitus as fasting blood glucose (BG) of ≥126 mg/dL, casual BG of ≥200 mg/dL, hemoglobin A1c of ≥6.5%, or using antidiabetic drugs; dyslipidemia as low-density lipoprotein cholesterol of ≥140 mg/dL, triglycerides of ≥150 mg/dL, high-density lipoprotein cholesterol of <40 mg/dL, or using antilipidemic drugs; and CKD as an estimated glomerular filtration rate (eGFR) of <60 mL/min/1.73 m², which was calculated using the estimation equation for Japanese patients with CKD [25].

The primary outcome was the change in estimated salt intake (g/day), calculated using the Tanaka formula [26]. Secondary outcomes included changes in office systolic blood pressure (SBP), diastolic blood pressure (DBP), body weight, BMI, and eGFR, as well as correlates of salt intake change.

Statistical analysis

Continuous variables are presented as mean ± SD. Pre- and post-campaign comparisons were performed using paired t-tests. Correlations between changes in salt intake and other variables were analyzed using restricted maximum likelihood (REML) estimation. Statistical analyses were performed using JMP (version 18.0.1; SAS Institute Inc., Cary, NC), with significance defined as p < 0.05.

## Results

**Figure 1:**
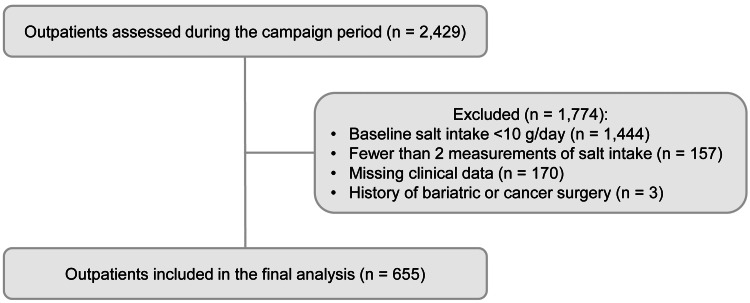
Flow diagram of patient selection for the analysis of the salt reduction campaign During the campaign period, 2,429 outpatients were assessed. Of these, 1,774 were excluded for the following reasons: baseline salt intake <10 g/day (n = 1,444), fewer than two measurements of salt intake (n = 157), missing clinical data (n = 170), and history of bariatric or cancer surgery (n = 3). A total of 655 outpatients were included in the final analysis.

A total of 655 patients were included in the final analysis, including 278 women (42.4%). The mean age was 61.6 ± 13.1 years. The baseline characteristics are shown in Table 1, and Table 2 presents the primary and secondary outcomes. Figure 1 presents the flow diagram of patient selection for the analysis of the salt reduction campaign. At baseline, mean salt intake was 11.6 ± 1.69 g/day. After the campaign, salt intake decreased significantly to 9.4 ± 2.44 g/day (p < 0.001). SBP decreased from 132.3 ± 13.0 to 131.1 ± 13.5 mmHg (p = 0.028), body weight from 70.6 ± 15.3 to 70.3 ± 15.2 kg (p < 0.001), and BMI from 27.4 ± 5.0 to 27.2 ± 5.0 kg/m² (p < 0.001). The DBP did not change significantly. The eGFR declined slightly from 67.3 ± 21.3 to 65.9 ± 21.2 mL/min/1.73 m² (p < 0.001). In subgroup analyses, among non-CKD patients, baseline eGFR was 78.4 ± 15.6 mL/min/1.73 m², and eGFR decreased slightly by 1.7 mL/min/1.73 m² after the campaign (p < 0.001). In contrast, among patients with CKD, baseline eGFR was 45.4 mL/min/1.73 m², and the change in eGFR was -0.7 mL/min/1.73 m², which was not statistically significant. No clinically significant renal events, including acute kidney injury, were observed during the study period. Correlation analysis showed that reductions in salt intake were weak but significantly associated with decreases in body weight (r = 0.183, p < 0.001), BMI (r = 0.192, p < 0.001), and SBP (r = 0.176, p < 0.001). Changes in salt intake were not associated with nutritional counseling by registered dietitians (r = 0.004, p = 0.914).

**Table 1:** Demographic and clinical characteristics of outpatients Data are presented as mean ± SD or number (%).

Variables	Values
Number	655
Age, years	61.6 ± 13.1
Female sex, n (%)	278 (42.4)
Body weight, kg	70.6 ± 15.3
Body mass index, kg/m²	27.4 ± 5.0
Systolic blood pressure, mmHg	132.3 ± 13.0
Diastolic blood pressure, mmHg	72.5 ± 11.3
Estimated salt intake, g/day	11.6 ± 1.69
Estimated glomerular filtration rate, mL/min/1.73 m²	67.3 ± 21.3
Obesity, n (%)	418 (63.8)
Hypertension, n (%)	486 (74.2)
Diabetes mellitus, n (%)	429 (65.5)
Dyslipidemia, n (%)	523 (79.8)
Chronic kidney disease, n (%)	184 (33.6)

**Table 2:** Effect of salt reduction campaign on clinical parameters Data are presented as mean ± SD.

Clinical parameters	Pre-campaign	Post-campaign	P-value
Estimated salt intake, g/day	11.6 ± 1.69	9.4 ± 2.44	< 0.001
Body weight, kg	70.6 ± 15.3	70.3 ± 15.2	< 0.001
Body mass index, kg/m²	27.4 ± 5.0	27.2 ± 5.0	< 0.001
Systolic blood pressure, mmHg	132.3 ± 13.0	131.1 ± 13.5	0.028
Diastolic blood pressure, mmHg	72.5 ± 11.3	71.8 ± 11.3	0.086
Estimated glomerular filtration rate, mL/min/1.73 m²	67.3 ± 21.3	65.9 ± 21.2	< 0.001

## Discussion

This study demonstrated that the clinic-based salt reduction campaign was associated with reductions in the estimated salt intake, SBP, body weight, and BMI of outpatients. Although eGFR showed a small decline, no clinically significant adverse renal events, such as acute kidney injury, were observed, suggesting that the observed changes were not clinically meaningful. In subgroup analyses, a small but statistically significant decline in eGFR was observed in non-CKD patients, whereas no significant change was observed in patients with CKD. These findings indicate that the observed changes in eGFR did not preferentially affect patients with impaired renal function. These data suggest the benefits of simple, clinic-based interventions in real-world practice.

Although several population-based salt reduction interventions have been implemented and evaluated, the clinic-based interventions remain understudied. A recent meta-analysis identified only four RCTs, with inconclusive findings regarding the effects of clinic- or facility-based salt reduction on salt intake and BP [17]. Our findings add to the limited evidence suggesting that clinic-based strategies can meaningfully contribute to salt intake reduction and BP control.

The antihypertensive effects of salt reduction are well established. A meta-analysis showed that a 4.4 g/day reduction in salt intake decreased BP by 4.2/2.1 mmHg in hypertensive individuals [10], and another review of 103 RCTs reported that a 2.3 g/day reduction reduced SBP by 3.8 mmHg [2], indicating an approximately 1 mmHg SBP reduction per 1 g/day decrease in salt intake. A larger meta-analysis of 133 RCTs confirmed a near-linear dose-response relationship [16]. In our study, salt intake decreased by 2.3 g/day, accompanied by a SBP reduction of 1.2 mmHg - smaller than previous estimates, likely reflecting the relatively well-controlled baseline BP (<140/90 mmHg). He et al. showed that greater BP reduction occurred in hypertensive individuals than in normotensive individuals following salt reduction [14]. Differences in salt intake assessment may also account for this variability. Although the gold standard for the evaluation of salt intake is 24-hour urine collection [6], we used spot urine, which correlates with 24-hour sodium excretion [1,26] and is convenient for repeated use in clinical settings despite diurnal variations and dietary influences [1,7,27]. Therefore, spot urine remains a practical tool for routine practice and is appropriate for this campaign.

Salt intake is also associated with weight gain and obesity. Previous studies reported a positive correlation between BMI and salt intake [28,29]. In our study, reductions in salt intake were correlated with decreases in body weight and BMI, suggesting that salt and weight reduction together enhance BP control, particularly among obese or hypertensive patients through reduced fluid retention [1,27]. Importantly, our findings imply that population-level educational measures, such as in-clinic displays, can facilitate behavioral change when combined with individualized advice.

Nakano et al. reported that intensive and frequent nutritional counseling by registered dietitians effectively reduced salt intake, office BP, and out-of-office BP in patients with hypertension [30]. In contrast, our study found no additional benefit of individualized counseling by registered dietitians, likely due to the limited frequency and duration, underscoring the need for sustained and structured counseling to promote long-term behavioral change.

Several limitations should be considered. First, due to the retrospective design, causal relationships cannot be established. Second, urine samples were collected under non-standardized conditions, which may have introduced variability. Third, salt intake was estimated using spot urine rather than 24-hour urine collection. Fourth, because patients were aware of the campaign, behavioral changes influenced by social desirability bias cannot be excluded. Fifth, information on changes in antihypertensive medications was not available. Finally, regression to the mean may have contributed to the observed changes. In addition, detailed information on the frequency of exposure to campaign materials was not available, and no questionnaire-based assessment of patient engagement or perceptions of the salt reduction campaign was conducted.

## Conclusions

This community clinic-based salt reduction campaign was associated with reductions in estimated salt intake and modest improvements in BP and body weight in routine clinical practice.

These findings suggest that simple educational and motivational strategies implemented in primary care settings may contribute to population-level salt reduction and support the management of hypertension.
